# Supplementary material for: HIV-1 Vpu is a potent transcriptional suppressor of NF-κB-elicited antiviral immune responses
Source: eLife. 2019 Feb 5;8:e41930. doi: 10.7554/eLife.41930 (PMC6372280; doi:10.7554/eLife.41930)
Supplement: Supplementary file 1. [file elife-41930-supp1.docx]

**Supplementary File 1: Identity and integrity of RNA-Seq samples.**

| **Sample ID** | **Donor (sex)** | **HIV-1 clone** | **Vpu status** | **RIN score** |
| --- | --- | --- | --- | --- |
| **1** | A  (male) | mock | N/A | 10.0 |
| **2** |  | CH293 | wild type | 10.0 |
| **3** |  |  | *vpu* stop | 10.0 |
| **4** |  |  | R50K | 10.0 |
| **5** |  |  | A20L/A24L | 10.0 |
| **6** |  | CH077 | wild type | 9.9 |
| **7** |  |  | *vpu* stop | 10.0 |
| **8** |  |  | R45K | 10.0 |
| **9** |  |  | A15L/A19L | 10.0 |
| **10** |  | STCO1 | wild type | 10.0 |
| **11** |  |  | *vpu* stop | 9.9 |
| **12** |  |  | R45K | 10.0 |
| **13** |  |  | A15L/A19L | 10.0 |
| **14** | B  (female) | mock | N/A | 10.0 |
| **15** |  | CH293 | wild type | 10.0 |
| **16** |  |  | *vpu* stop | 10.0 |
| **17** |  |  | R50K | 10.0 |
| **18** |  |  | A20L/A24L | 10.0 |
| **19** |  | CH077 | wild type | 10.0 |
| **20** |  |  | *vpu* stop | 10.0 |
| **21** |  |  | R45K | 10.0 |
| **22** |  |  | A15L/A19L | 10.0 |
| **23** |  | STCO1 | wild type | 10.0 |
| **24** |  |  | *vpu* stop | 10.0 |
| **25** |  |  | R45K | 10.0 |
| **26** |  |  | A15L/A19L | 10.0 |
| **27** | C  (male) | mock | N/A | 10.0 |
| **28** |  | CH293 | wild type | 10.0 |
| **29** |  |  | *vpu* stop | 10.0 |
| **30** |  |  | R50K | 10.0 |
| **31** |  |  | A20L/A24L | 10.0 |
| **32** |  | CH077 | wild type | 10.0 |
| **33** |  |  | *vpu* stop | 10.0 |
| **34** |  |  | R45K | 10.0 |
| **35** |  |  | A15L/A19L | 8.6 |
| **36** |  | STCO1 | wild type | 10.0 |
| **37** |  |  | *vpu* stop | 10.0 |
| **38** |  |  | R45K | 10.0 |
| **39** |  |  | A15L/A19L | 10.0 |
| **40** | D  (female) | mock | N/A | 10.0 |
| **41** |  | CH293 | wild type | 10.0 |
| **42** |  |  | *vpu* stop | 10.0 |
| **43** |  |  | R50K | 10.0 |
| **44** |  |  | A20L/A24L | 10.0 |
| **45** |  | CH077 | wild type | 10.0 |
| **46** |  |  | *vpu* stop | 10.0 |
| **47** |  |  | R45K | 10.0 |
| **48** |  |  | A15L/A19L | 10.0 |
| **49** |  | STCO1 | wild type | 10.0 |
| **50** |  |  | *vpu* stop | 10.0 |
| **51** |  |  | R45K | 10.0 |
| **52** |  |  | A15L/A19L | 10.0 |
