# Supplementary material for: HIV-1 Vpu is a potent transcriptional suppressor of NF-κB-elicited antiviral immune responses
Source: eLife. 2019 Feb 5;8:e41930. doi: 10.7554/eLife.41930 (PMC6372280; doi:10.7554/eLife.41930)
Supplement: Supplementary file 2. [file elife-41930-supp2.docx]

**Supplementary File 2: Custom-defined gene set of NF-κB target genes.**

| **Gene symbol** | **Gene title** |
| --- | --- |
| *AGER* | advanced glycosylation end-product specific receptor |
| *ALOX12B* | arachidonate 12-lipoxygenase, 12R type |
| *APOC3* | apolipoprotein C3 |
| *BCL2* | BCL2, apoptosis regulator |
| *BCL2A1* | BCL2 related protein A1 |
| *BCL2L1* | BCL2 like 1 |
| *BCL3* | B cell CLL/lymphoma 3 |
| *BDKRB1* | bradykinin receptor B1 |
| *CXCR5* | C-X-C motif chemokine receptor 5 |
| *B2M* | beta-2-microglobulin |
| *NOD2* | nucleotide binding oligomerization domain containing 2 |
| *CCL11* | C-C motif chemokine ligand 11 |
| *CCL15* | C-C motif chemokine ligand 15 |
| *CCL2* | C-C motif chemokine ligand 2 |
| *CCL5* | C-C motif chemokine ligand 5 |
| *CCND1* | cyclin D1 |
| *CCR5* | C-C motif chemokine receptor 5 (gene/pseudogene) |
| *CCR7* | C-C motif chemokine receptor 7 |
| *ENG* | endoglin |
| *CD209* | CD209 molecule |
| *CD3G* | CD3g molecule |
| *CD44* | CD44 molecule (Indian blood group) |
| *CD48* | CD48 molecule |
| *CD69* | CD69 molecule |
| *CD74* | CD74 molecule |
| *CD80* | CD80 molecule |
| *CD83* | CD83 molecule |
| *CR2* | complement C3d receptor 2 |
| *CRP* | C-reactive protein |
| *CSF1* | colony stimulating factor 1 |
| *CSF2* | colony stimulating factor 2 |
| *CSF3* | colony stimulating factor 3 |
| *CXCL5* | C-X-C motif chemokine ligand 5 |
| *DEFB4A* | defensin beta 4A |
| *ELF3* | E74 like ETS transcription factor 3 |
| *F3* | coagulation factor III, tissue factor |
| *GSTP1* | glutathione S-transferase pi 1 |
| *PSMA2* | proteasome subunit alpha 2 |
| *HMOX1* | heme oxygenase 1 |
| *ICAM1* | intercellular adhesion molecule 1 |
| *IER3* | immediate early response 3 |
| *IFNB1* | interferon beta 1 |
| *IGHG3* | immunoglobulin heavy constant gamma 3 (G3m marker) |
| *IGHG4* | immunoglobulin heavy constant gamma 4 (G4m marker) |
| *IL11* | interleukin 11 |
| *IL13* | interleukin 13 |
| *IL15* | interleukin 15 |
| *IL15RA* | interleukin 15 receptor subunit alpha |
| *IL1A* | interleukin 1 alpha |
| *IL1B* | interleukin 1 beta |
| *IL1RN* | interleukin 1 receptor antagonist |
| *IL2* | interleukin 2 |
| *IL2RA* | interleukin 2 receptor subunit alpha |
| *IL6* | interleukin 6 |
| *CXCL8* | C-X-C motif chemokine ligand 8 |
| *IL9* | interleukin 9 |
| *IRF1* | interferon regulatory factor 1 |
| *IRF2* | interferon regulatory factor 2 |
| *IRF4* | interferon regulatory factor 4 |
| *IRF7* | interferon regulatory factor 7 |
| *KLK3* | kallikrein related peptidase 3 |
| *PSMB9* | proteasome subunit beta 9 |
| *LTA* | lymphotoxin alpha |
| *LTB* | lymphotoxin beta |
| *MMP9* | matrix metallopeptidase 9 |
| *MYC* | MYC proto-oncogene, bHLH transcription factor |
| *NFKB1* | nuclear factor kappa B subunit 1 |
| *NFKB2* | nuclear factor kappa B subunit 2 |
| *NFKBIA* | NFKB inhibitor alpha |
| *NOS2* | nitric oxide synthase 2 |
| *NQO1* | NAD(P)H quinone dehydrogenase 1 |
| *NR4A2* | nuclear receptor subfamily 4 group A member 2 |
| *OPRM1* | opioid receptor mu 1 |
| *PDGFB* | platelet derived growth factor subunit B |
| *PLAU* | plasminogen activator, urokinase |
| *PLCD1* | phospholipase C delta 1 |
| *PTAFR* | platelet activating factor receptor |
| *PTGS2* | prostaglandin-endoperoxide synthase 2 |
| *PTX3* | pentraxin 3 |
| *RELB* | RELB proto-oncogene, NF-κB subunit |
| *S100A6* | S100 calcium binding protein A6 |
| *SCNN1A* | sodium channel epithelial 1 alpha subunit |
| *SELE* | selectin E |
| *SELP* | selectin P |
| *SLC2A5* | solute carrier family 2 member 5 |
| *SOD2* | superoxide dismutase 2 |
| *STAT5A* | signal transducer and activator of transcription 5A |
| *TACR1* | tachykinin receptor 1 |
| *TAP1* | transporter 1, ATP binding cassette subfamily B member |
| *TFPI2* | tissue factor pathway inhibitor 2 |
| *TGM2* | transglutaminase 2 |
| *TNC* | tenascin C |
| *TNF* | tumor necrosis factor |
| *TNFAIP3* | TNF alpha induced protein 3 |
| *CD40* | CD40 molecule |
| *FAS* | Fas cell surface death receptor |
| *TNFRSF9* | TNF receptor superfamily member 9 |
| *CD40LG* | CD40 ligand |
| *FASLG* | Fas ligand |
| *TP53* | tumor protein p53 |
| *TPMT* | thiopurine S-methyltransferase |
| *VCAM1* | vascular cell adhesion molecule 1 |
| *VEGFC* | vascular endothelial growth factor C |
| *VIM* | vimentin |
