# Supplementary material for: HIV-1 Vpu is a potent transcriptional suppressor of NF-κB-elicited antiviral immune responses
Source: eLife. 2019 Feb 5;8:e41930. doi: 10.7554/eLife.41930 (PMC6372280; doi:10.7554/eLife.41930)
Supplement: Supplementary file 3. [file elife-41930-supp3.docx]

**Supplementary File 3: Custom-defined gene set of host restriction factors.**

| **Gene symbol** | **Gene title** |
| --- | --- |
| *APOBEC3A* | apolipoprotein B mRNA editing enzyme, catalytic polypeptide-like 3A |
| *APOBEC3B* | apolipoprotein B mRNA editing enzyme, catalytic polypeptide-like 3B |
| *APOBEC3C* | apolipoprotein B mRNA editing enzyme, catalytic polypeptide-like 3C |
| *APOBEC3D* | apolipoprotein B mRNA editing enzyme, catalytic polypeptide-like 3D |
| *APOBEC3F* | apolipoprotein B mRNA editing enzyme, catalytic polypeptide-like 3F |
| *APOBEC3G* | apolipoprotein B mRNA editing enzyme, catalytic polypeptide-like 3G |
| *APOBEC3H* | apolipoprotein B mRNA editing enzyme, catalytic polypeptide-like 3H |
| *BST2* | Bone marrow stromal antigen 2 |
| *CH25H* | cholesterol 25-hydroxylase |
| *GBP5* | guanylate binding protein 5 |
| *HERC5* | hect domain and RLD 5 |
| *IFI16* | interferon, gamma-inducible protein 16 |
| *IFIT1* | interferon-induced protein with tetratricopeptide repeats 1 |
| *IFIT2* | interferon-induced protein with tetratricopeptide repeats 2 |
| *IFIT3* | interferon-induced protein with tetratricopeptide repeats 3 |
| *IFIT5* | interferon-induced protein with tetratricopeptide repeats 5 |
| *IFITM1* | interferon induced transmembrane protein 1 (9-27) |
| *IFITM2* | interferon induced transmembrane protein 2 |
| *IFITM3* | interferon induced transmembrane protein 3 (1-8U) |
| *ISG15* | ISG15 ubiquitin-like modifier |
| *LGALS3BP* | lectin, galactoside-binding, soluble, 3 binding protein |
| *MX1* | myxovirus (influenza virus) resistance 1, interferon-inducible protein p78 |
| *MX2* | myxovirus (influenza virus) resistance 2 |
| *OAS1* | 2',5'-oligoadenylate synthetase 1, 40/46kDa |
| *EIF2AK2* | eukaryotic translation initiation factor 2-alpha kinase 2 |
| *RNASEL* | ribonuclease L (2',5'-oligoisoadenylate synthetase-dependent) |
| *SAMHD1* | SAM domain and HD domain 1 |
| *SERINC3* | serine incorporator 3 |
| *SERINC5* | serine incorporator 5 |
| *SLFN11* | schlafen family member 11 |
| *TRIM5* | tripartite motif-containing 5 |
| *TRIM28* | tripartite motif-containing 28 |
| *RSAD2* | radical S-adenosyl methionine domain containing 2 |
| *ZC3HAV1L* | zinc finger CCCH-type, antiviral 1-like |
