# Supplementary material for: HIV-1 Vpu is a potent transcriptional suppressor of NF-κB-elicited antiviral immune responses
Source: eLife. 2019 Feb 5;8:e41930. doi: 10.7554/eLife.41930 (PMC6372280; doi:10.7554/eLife.41930)
Supplement: Supplementary file 4. [file elife-41930-supp4.docx]

**Supplementary File 4: Primers used for mutagenesis of *vpu***

| HIV-1 mutant | Primer name | Primer sequence (5’ – 3’) |
| --- | --- | --- |
| CH293 *vpu* stop | CH293 *vpu* stop fw | ATCTTATATCAAAGCAGTAAGTACTAAGTAGTATATGTAATGTAATGATGGATAGAAAAAGCAGATTATA |
|  | CH293 *vpu* stop rev | TATAATCTGCTTTTTCTATCCATCATTACATTACATATACTACTTAGTACTTACTGCTTTGATATAAGAT |
| CH293 Vpu R50K | CH293 R50K fw | GTTAAAAAAATTAGGGAAAGAGC |
|  | CH293 R50K rev | GCTCTTTCCCTAATTTTTTTAAC |
| CH293 Vpu A20L/A24L | CH293 A20L/A24L fw | GCATTGGTAGTACTAATAATTATACTAATAATTGTGTGG |
|  | CH293 A20L/A24L rev | CCACACAATTATTAGTATAATTATTAGTACTACCAATGC |
| CH077 *vpu* stop | CH077 *vpu* stop fw | CTACCAAAGCAGTAAGTAGCATCTGTAATGTAATGATTATATATATTAGGAATAGTAGCATTAGTA |
|  | CH077 *vpu* stop rev | TACTAATGCTACTATTCCTAATATATATAATCATTACATTACAGATGCTACTTACTGCTTTGGTAG |
| CH077 Vpu R45K | CH077 R45K fw | CAGATTACTTGATAAAATAATAGACAGAGC |
|  | CH077 R45K rev | GCTCTGTCTATTATTTTATCAAGTAATCTG |
| CH077 Vpu A15L/A19L | CH077 A15L/A19L fw | CAATATTACTAATAGTTGTGTGGACCATAGTATACATAG |
|  | CH077 A15L/A19L rev | CCACACAACTATTAGTAATATTGCTAGTACTAC |
| STCO1 *vpu* stop | STCO1 *vpu* stop fw | CTACCAAAGCAGTAAGTAGTATATGTAATGTAATGATTGCATATAGCAGCAATAGTAGGATTAGTA |
|  | STCO1 *vpu* stop rev | TACTAATCCTACTATTGCTGCTATATGCAATCATTACATTACATATACTACTTACTGCTTTGGTAG |
| STCO1 Vpu R45K | STCO1 R45K fw | GCTAATTGAAAAAATAAGTGAAAGAGC |
|  | STCO1 R45K rev | GCTCTTTCACTTATTTTTTCAATTAGC |
| STCO1 Vpu A15L/A19L | STCO1 A15L/A19L fw | GGATTAGTAGTACTAGCAATACTACTAATAGTTGTGTG |
|  | STCO1 A15L/A19L rev | CACACAACTATTAGTAGTATTGCTAGTACTACTAATCC |
