# Supplementary material for: HIV-1 Vpu is a potent transcriptional suppressor of NF-κB-elicited antiviral immune responses
Source: eLife. 2019 Feb 5;8:e41930. doi: 10.7554/eLife.41930 (PMC6372280; doi:10.7554/eLife.41930)
Supplement: Transparent reporting form [file elife-41930-transrepform.pdf]

1st Floor
24 Hills Road
Cambridge CB2 1JP, UK

P 01223 855340
W elifesciences.org
T @elife

eLife's transparent reporting form

We encourage authors to provide detailed information *within their submission* to facilitate the interpretation and replication of experiments. Authors can upload supporting documentation to indicate the use of appropriate reporting guidelines for health-related research (see EQUATOR Network), life science research (see the BioSharing Information Resource), or the ARRIVE guidelines for reporting work involving animal research. Where applicable, authors should refer to any relevant reporting standards documents in this form.

If you have any questions, please consult our Journal Policies and/or contact us: editorial@elifesciences.org.

Sample-size estimation

- You should state whether an appropriate sample size was computed when the study was being designed
- You should state the statistical method of sample size computation and any required assumptions
- If no explicit power analysis was used, you should describe how you decided what sample (replicate) size (number) to use

Please outline where this information can be found within the submission (e.g., sections or figure legends), or explain why this information doesn't apply to your submission:

No explicit power analysis was used. In RNA-Seq studies, three independent samples are frequently analyzed. We used four samples (2 female, 2 male donors) to increase statistical power and to identify possible outliers. Furthermore, we validated the relevance of our findings using three different HIV-1 clones (i.e. CH077, STCO1 and CH293) (see lines 523-540). For phenotypic characterization of the HIV-1 clones, at least three independent experiments were performed. Both, Vpu-mediated tetherin counteraction and inhibition of NF-κB activation were confirmed using two independent experimental approaches.

Replicates

- You should report how often each experiment was performed
- You should include a definition of biological versus technical replication
- The data obtained should be provided and sufficient information should be provided to indicate the number of independent biological and/or technical replicates
- If you encountered any outliers, you should describe how these were handled
- Criteria for exclusion/inclusion of data should be clearly stated
- High-throughput sequence data should be uploaded before submission, with a private link for reviewers provided (these are available from both GEO and ArrayExpress)

Please outline where this information can be found within the submission (e.g., sections or figure legends), or explain why this information doesn't apply to your submission:

eLife Sciences Publications, Ltd is a limited liability non-profit non-stock corporation incorporated in the State of Delaware, USA, with company number 5030732, and is registered in the UK with company number FC030576 and branch number BR015634 at the address 1st Floor, 24 Hills Road, Cambridge CB2 1JP | August 2014

The number of biological replicates is stated in the respective figure legends (lines 840/841, 845/846, 851/852, 858/859, 890, 899/900, 907-910, 913-915, 936/937, 946/947, 952/954, 956, 985/986) and the materials and methods section (lines 524-526). Independent experiments are defined as infections of cells from different donors or transfections performed on different days. As stated in the manuscript (e.g. lines 413, 464, 505), each independent qPCR, TZM-bl and NF-κB luciferase reporter experiment was performed in technical replicates.
No outliers were detected or omitted (lines 533/534). In Fig. 4C, cytokine concentrations that were below the detection limit in more than one donor were omitted from the analyses (lines 915/916). As stated in lines 552-554 and the Key Resources Table, all RNA sequencing data have been uploaded to the Gene Expression Omnibus(GEO) database (accession number #GSE117655). To access the data, go to https://www.ncbi.nlm.nih.gov/geo/query/acc.cgi?acc=GSE117655 and enter token ktqnsqcqftgvruf.

Statistical reporting

- Statistical analysis methods should be described and justified
- Raw data should be presented in figures whenever informative to do so (typically when N per group is less than 10)
- For each experiment, you should identify the statistical tests used, exact values of N, definitions of center, methods of multiple test correction, and dispersion and precision measures (e.g., mean, median, SD, SEM, confidence intervals; and, for the major substantive results, a measure of effect size (e.g., Pearson's r, Cohen's d)
- Report exact p-values wherever possible alongside the summary statistics and 95% confidence intervals. These should be reported for all key questions and not only when the p-value is less than 0.05.

Please outline where this information can be found within the submission (e.g., sections or figure legends), or explain why this information doesn't apply to your submission:

Statistical analyses are described and justified in the materials and methods section, including methods for multiple comparison (lines 523-546). The number of biological replicates (N) and precision measures (mean, SEM) are indicated in each figure legend. Individual data points/raw data are shown in Figure 1F, Figure 1-figure supplement 1B, 2A, Figure 2C, Figure 2-figure supplement 1A, Figure 3A, Figure 3-figure supplement 1A, Figure 4B. Individual data points are not shown for large N (e.g. Fig. 4C). Exact p values are shown in the bottom panels of Fig. 4A, but were replaced by asterisks due to space limitations in other figures (*** p< 0.001; ** p<0.01; * p<0.05).

(For large datasets, or papers with a very large number of statistical tests, you may upload a single table file with tests, Ns, etc., with reference to sections in the manuscript.)

Group allocation

- Indicate how samples were allocated into experimental groups (in the case of clinical studies, please specify allocation to treatment method); if randomization was used, please also state if restricted randomization was applied
- Indicate if masking was used during group allocation, data collection and/or data analysis

Please outline where this information can be found within the submission (e.g., sections or figure legends), or explain why this information doesn't apply to your submission:

Group allocation does not apply to this submission since CD4+ T cells from all donors were infected with the same wild type and mutant HIV-1 variants (e.g. lines 523-526).

Additional data files ("source data")

- We encourage you to upload relevant additional data files, such as numerical data that are represented as a graph in a figure, or as a summary table
- Where provided, these should be in the most useful format, and they can be uploaded as "Source data" files linked to a main figure or table
- Include model definition files including the full list of parameters used
- Include code used for data analysis (e.g., R, MatLab)
- Avoid stating that data files are "available upon request"

Please indicate the figures or tables for which source data files have been provided:

No source data files have been provided. The RNA-Seq data have been uploaded to the GEO database (accession number #GSE117655) (lines 482-486). RNA integrity numbers (RIN scores) of all samples are listed in Supplementary File 1.
